# Quality of life in patients with personality disorders seen at an ordinary psychiatric outpatient clinic

**DOI:** 10.1186/1471-244X-5-10

**Published:** 2005-02-20

**Authors:** Kjersti Narud, Arnstein Mykletun, Alv A Dahl

**Affiliations:** 1 Department of Psychiatry, Aker University Hospital, Sognsvannsveien 21, 0310 Oslo, Norway; 2 Center for Health Promotion, Faculty of Psychology, University of Bergen Christiesgate 13, 5015 Bergen, Norway; 4 Department of Clinical Cancer Research, Rikshospitalet-Radiumhospitalet Trust, Montebello, 0310 Oslo, Norway

## Abstract

**Background:**

Epidemiological studies have found reduced health-related quality of life (QoL) in patients with personality disorders (PDs), but few clinical studies have examined QoL in PDs, and none of them are from an ordinary psychiatric outpatient clinic (POC). We wanted to examine QoL in patients with PDs seen at a POC, to explore the associations of QoL with established psychiatric measures, and to evaluate QoL as an outcome measure in PD patients.

**Methods:**

72 patients with PDs at a POC filled in the MOS Short Form 36 (SF-36), and two established psychiatric self-rating measures. A national norm sample was compared on the SF-36. An independent psychiatrist diagnosed PDs and Axis-I disorders by structured interviews and rated the Global Assessment of Functioning (GAF). All measurements were repeated in the 39 PD patients that attended the 2 years follow-up examination.

**Results:**

PD patients showed high co-morbidity with other PDs and Axis I mental disorders, and they scored significantly lower on all the SF-36 dimensions than age- and gender-adjusted norms. Adjustment for co-morbid Axis I disorders had some influence, however. The SF-36 mental health, vitality, and social functioning were significantly associated with the GAF and the self-rated psychiatric measures. Significant changes at follow-up were found in the psychiatric measures, but only on the mental health and role-physical of the SF-36.

**Conclusion:**

Patients with PDs seen for treatment at a POC have globally poor QoL. Both physical and mental dimensions of the SF-36 are correlated with established psychiatric measures in such patients, but significant changes in these measures are only partly associated with changes in the SF-36 dimensions.

## Background

According to the DSM-IV [1] personality disorders (PDs) are characterized by enduringly deviating patterns of perceiving, relating to, and thinking about the environment and oneself that are exhibited in a wide range of social and personal contexts. Such patterns lead to "clinically significant distress or impairment in social, occupational, or other important areas of functioning". The DSM-IV does not indicate how "clinically significant distress or impairment" (page 633) should be evaluated, however, and a recent study showed that various formulations of this criterion hardly increased diagnostic validity [2].

Since the DSM-IV included the Global Assessment of Functioning Scale (GAF) as Axis V, it is reasonable to consider if the GAF should be used for evaluations of "significant distress or impairment". Two problems are implicit in such an approach: at what GAF cut-off score should "significant distress or impairment" be set, and the evaluation is only done by a professional. As to the first problem, Kessler et al [3] suggested a GAF score of 60 as a cut off for "serious mental illness". As to the second, in patients with somatic diseases "clinically significant distress or impairment" has for a long time been quantified by self-rating of the health-related quality of life (QoL) including mental health [4]. Several general instruments for the rating of QoL have been developed among which the Medical Outcome Study Short Form-36 (the SF-36) and its brief version the SF-12 have become the most popular [5,6].

Major treatment outcome studies of PDs like the Collaborative Longitudinal Personality Disorders Study [7], the Norwegian Network of Psychotherapeutic Day Hospitals [8], and the Cassel Hospital study [9] used the GAF, however, and did not included any QoL measurements, and the QoL was not included in a recommended core battery of instruments for measurement of changes in PD patients [10].

In contrast, two epidemiological studies of PDs have used the SF-12 as a measure of disability. In a national study from Australia, Jackson and Burgess [11] reported that the SF-12 Physical and Mental Component Summary Scales (PCS and MCS) were both significantly reduced in persons with one or more PDs diagnosed by a screening instrument, compared to persons without. When further examining the relationship between the SF-12 and PDs, they found that co-morbid Axis I and chronic physical conditions explained a considerable part of the MCS and PCS scores in PDs [12]. Mean MCS and PCS scores also became significantly lower with an increasing number of comorbid PDs present. A national study from the United States [13] confirmed the reduced MCS after controlling for co-morbid Axis I disorder in the avoidant, dependent, paranoid, schizoid, and antisocial PDs, but not in the histrionic PD, diagnosed by a more extensive diagnostic interview schedule than the Australian study.

A review of the literature showed that the QoL had only been used as a disability measure in a few clinical research studies of PD patients. In depressed elderly patients, Abrams et al [14] found that the presence of criteria for cluster B PDs predicted lower QoL. Since the cluster B criteria overlapped considerably with symptoms of depression, it was unclear if they made any independent contribution to reduced QoL. Swinton et al [15] reported that male PD patients in a high security forensic setting were less satisfied with their overall QoL than patients with schizophrenia. The authors emphasized that the high security setting was quite unusual. Hueston et al. [16] showed that primary care patients with high risk for PDs, scored significantly lower on overall QoL and on several subscales of the SF-36, compared to patients with a low risk for PDs. Since prevalence of depression and alcohol dependence was higher in the high-risk group, the influence of PDs alone on QoL was difficult to tease out in that study. Nakao et al [17] examined the relationship between PDs and the GAF in 136 Axis I patients mainly with mood and anxiety disorders and found that patients with any comorbid PDs were more disabled than those without. They did not adjust for the presence of Axis I disorders, however.

None of these clinical studies takes QoL as observed in PD patients seen at an ordinary psychiatric outpatient clinic (POC) as their point of departure. However PD patients are frequent at POC, and the QoL is an important self-rated measure of "clinically significant distress or impairment". Since QoL data on PD patients seen at at POC seems to be lacking from the literature, we found it relevant to study a consecutive sample of PD patients from a POC and collect QoL data with SF-36. We posed the following research questions: 1) How are the SF-36 dimensions mean scores in PD patients compared to age- and gender-adjusted norm data? 2) To what extent are the SF-36 scores in PD patients associated with co-morbid Axis I disorders? 3) How is the association between the SF-36 dimension and established patient- and professional-rated psychiatric measures in PD patients? and 4) What changes in the SF-36 dimensions of treated PD patients are observed from baseline to follow-up, and how are they related to changes in the psychiatric measures?

## Methods

### Setting

Furuset POC serves a communality of Oslo City, Norway with a population of 28.000 people. At the time of the study, the staff consisted of three psychiatrists, three clinical psychologists, two psychiatric nurses, and two social workers. The intake rate was approximately 400 new patients a year. The first author (KN) invited the staff to take part in the study by referring to her new patients with probable PDs. Six professionals were willing to participate, while four declined due to heavy clinical burden, or lack of interest.

At the start of the study in 1996, Furuset was a new suburb of Oslo, and the inhabitants were characterized by lower socio-economic conditions, high mobility, and a considerable prevalence of immigrants from Asian countries. The suburb had a high proportion of municipal housings, and the criterion for allotment to them was severe mental disorder and/or severe socio-economic problems. Many patients seen at Furuset POC were out of work due to mental disorders, and/or due to socio-economic circumstances.

### Patients

Patients aged from 18 to 75 years were consecutively recruited from January 1, 1996 to June 30, 1998. The patients were referred from the local GPs, and physical examination and adequate treatment and follow-up of physical diseases were the responsibility of the GPs. The six therapists screened for probable PDs among new patients scheduled for treatment. Exclusion criteria were mental retardation, lifetime psychosis and bipolar disorder, organic mental disorders, current strong suicidal ideation, and insufficient knowledge of the Norwegian language. Eligible patients received oral and written information about the study from their therapists. Then the patients were invited to take part in the study, and they all gave written informed consent. The Ethical Review Board of Department of Psychiatry, Aker University Hospital approved the project.

The six therapists did not miss out any patients at screening, but 5 (4%) eligible patients declined to take part in the study. Among 110 eligible patients referred to the study, only 91 filled in the SF-36 at baseline due to administrative misunderstandings. However, when they were compared to the 19 who did not fill in, the non-attenders only had significantly fewer co-morbid Axis I-disorders (data not shown).

In order to answer the research questions, the sample was divided into three groups: cluster A+B PDs (n = 39), cluster C (n = 33), and Axis I-disorders (n = 19). The cluster A+B group could also contain co-morbid cluster C PDs and Axis I-disorders, and the cluster C co-morbid Axis I-disorders.

### Follow-up procedure

Two years after baseline, the patients received a mailed written appointment for a follow-up interview. Those who did not show up were sent a written reminder. If they still did not meet, they were called by phone, and if there was no answer, their addresses and phone numbers were checked at the Census register. Appointments were mailed to new addresses, and phone-calls were made in case of non-response. Only a few patients responded to these extended search procedures.

### Norm sample

Norm data on the SF-36 was obtained from the Survey of Level of Living in Norway 1998 [18] comprising 6.638 participants aged 23 to 75 years. The norm data were adjusted by gender and distribution into 5-year age groups in relation to the PD sample.

### Assessments

At baseline, diagnoses of PDs were made with the use of the Personality Disorder Examination, and Axis I-disorders were diagnosed by the MINI-International Neuropsychiatric Interview. Anamnestic data were collected, and global assessment of function was rated. The professional-based interviews and examinations of all patients at baseline and follow-up were carried out by a single experienced psychiatrist (KN), who did not take part in any treatment given.

All patients also filled in the following self-rating instruments at baseline: the SF-36, the Social Adjustment Scale, and the Symptom Checklist 90-Revised Personality Severity Index.

At follow-up all these assessments were repeated, and additional information about treatment as well as job/education, social- and family changes was collected.

### Measures

#### Professional-rated

The *Personality Disorder Examination *(PDE) [19] is a structured clinical interview for PDs according to the DSM-III-R with good inter-rater reliability, and wide international application. Findings are reported as PD diagnoses, and as dimensional PD scores based on the sum of the scoring on each PD criterion (0: not present, 1: probably present, and 2: definitely present). Dimensional scores for the PD clusters are used as a main psychopathology variable, and the numbers of PDs are also reported.

The *MINI International Neuropsychiatric Interview *[20] was used to diagnose Axis-I disorders according to DSM-IV. The MINI covers 18 Axis-I disorders, has been translated into many languages and has demonstrated good inter-rater reliability. Findings are reported as numbers and percentages of patients with positive Axis-I diagnoses, and as mean number of such diagnoses.

The *Global Assessment of Functioning (GAF) *is a rating scale for the current evaluation of the overall functioning of a subject on a continuum from severe mental disorder to complete mental health that was defined as Axis V of the DSM-IV. Scale values range from 1 (sickest individual) to 100 (the healthiest person). The scale is divided in ten equal intervals from 1 – 10 to 91 – 100. Most outpatients will be rated between 40 and 70, although some individuals rated above 70 may seek therapy. The GAF is a reliable instrument [21], and the cut-off score for 'minimal impairment' has been set at 70 points or higher [22] and for 'serious mental disorder' at lower than 60 [3].

#### Patient-rated

The *SF-36 *[5] was chosen for measurement of health-related QoL, since it is in widespread use, and has shown good psychometric properties in Norway [23]. The SF-36 has demonstrated sensitivity to change, and score changes can be interpreted as changes in the health-related quality of life of the patient. The SF-36 assesses eight dimensions of physical and mental health, and the range is from 100 (optimal) to zero (poorest): physical functioning (PF), physical role functioning (RP), bodily pain (BP), general health (GH), vitality (VT), social functioning (SF), emotional role functioning (RE), and mental health (MH).

The *Social Adjustment Scale *(SAS-SR) [24] contains 42 questions which investigate expressive or instrumental roles in six major areas of functioning: work, social and leisure activities, relationship with extended family, role as a spouse/ partner, role as a parent, and role as member of the family unit. Each area is measured as a continuous variable, and the scales for individual items range from 1 (best) to 5 (worst). The scores within each role area are summed, and a mean for each area is obtained. By adding up the scores of all items and dividing by the number of items actually scored, an overall adjustment score is obtained.

The *Symptom Checklist 90-Revised (SCL-90-R), Personality Severity Index *(PSI). The SCL-90-R is a 90-item self-report inventory assessing current levels of mental symptoms patterns. Each item is a description of a mental symptom rated on a five-point scale, and rates the degree of 'distress/discomfort' during the last week prior to its administration. Several indices based on the SCL-90-R scores have been defined, and the PSI is the mean value of 22 items covering the interpersonal sensitivity, anger/ hostility, and paranoid ideation subscales. The PSI reflects the presence and severity of relatively enduring characteristics of the patient, and is, therefore, relevant for the evaluation of severe PDs [25]. For the PSI, pathology is defined by a cut-off score of ≥ 1.0.

### Statistics

Data were analyzed by SPSS version 12.0. Descriptive statistics were conducted with independent and paired-sample t-test as well as one-way ANOVA (with Bonferroni's correction for multiple comparisons) for metric variables, and with χ^2 ^and Fisher's Exact Test for categorical variables. The Mann-Whitney test and the Wilcoxon signed-rank test were applied for metric variables when the data distribution violated parametric assumptions. Spearman's rank correlation was used for associations. One-sample t-tests were applied when the SF-36 dimension scores of the age- and gender-adjusted norm groups were compared to the means of the three diagnostic groups. The comparison of mean scores on the SF-36 dimensions between the three groups was conducted with oneway ANOVA with Bonferroni's correction. The influence on the QoL scores of patients with PDs of Axis I disorders and of cluster C PDs in the cluster A+B PDs group, was examined with linear regression analyses. All tests were two-sided, and the level of significance was set at p < .05.

## Results

### Sample characteristics

All the 91 patients included were Caucasian, and 48 (53%) were females. The mean age of the sample at baseline was 36.3 years (SD 10.5) and ranging from 19 to 74 years, with a median of 35 years. None of the patients had any significant somatic diseases as reported by their GPs.

Patients belonging to the cluster A+B, cluster C, and Axis I- disorders groups did not differ at baseline on demographic variables (Table 1).

**Table 1:** Demographic and psychopathological features at baseline in patients with cluster A+B, and cluster C personality disorders, and non-psychotic Axis I disorders.

**Variables**	**Cluster A+B **(n = 39)	**Cluster C **(n = 33)	**Axis I **(n = 19)	**p**
Age (mean, SD)	35.1 (11.2)	36.6 (10.9)	41.1 (10.4)	.15

Gender (n, %)				.74
Male	17 (44)	17 (52)	8 (42)	
Female	22 (55)	16 (48)	11 (58)	

Relationship (n, %)				.53
Paired	23 (59)	18 (55)	7 (37)	
Non-paired	16 (41)	15 (45)	12 (63)	

Basic education level (n, %)				.28
≤ 9 years	15 (33)	13 (32)	8 (33)	
10 – 12 years	14 (31)	16 (39)	13 (54)	
≥ 13 years	16 (36)	12 (29)	3 (13)	

Early childhood loss (n, %)	12 (31)	7 (21)	1 (5)	.22

Childhood sexual abuse (n, %)	7 (18)	7 (21)	1 (5)	.22

Age of onset mental problems (mean, SD)	16.7 (8.9)	20.0 (8.4)	27.0 (11.8)	<. 001 A+B, C vs Axis I

Work income (n, %)	23 (59)	21 (64)	13 (68)	.78

Economic support	16 (41)	12 (36)	6 (32)	

Income last year (1.000 NOK) (mean, SD)	154 (74)	178 (70)	179 (62)	.27

On sickleave last year (n, %)				.37
No	18 (46)	10 (30)	5 (26)	
≤ 12 weeks	11 (28)	12 (37)	5 (26)	
≥ 13 weeks	10 (26)	11 (33)	9 (52)	

No of PD cluster criteria (mean, SD)				
Total	41.8 (16.2)	26.6 (11.9)	8.1 (9.2)	< .001 A+B vs C vs Axis I
Cluster A	11.3 (8.2)	5.6 (4.6)	1.6 (2.0)	< .001 A+B vs C, Axis I
Cluster B	14.7 (10.9)	4.8 (5.4)	2.6 (3.7)	< .001 A+B vs C, Axis I
Cluster C	15.8 (10.0)	16.2 (5.7)	3.9 (5.4)	< .001 A+B, C vs Axis I

Comorbid Axis I disorder (n, %				.60
No	10 (26)	8 (24)	-	
Yes	29 (74)	25 (76)		

GAF (mean, SD)	41.5 (9.1)	51.0 (7.4)	55.4 (9.3)	< .001 A+B vs C, Axis I

PSI (mean, SD)	1.80 (.78)	1.24 (.71)	1.24 (.81)	.005 A+B vs C, axis I

SAS (mean, SD) Overall adjustment	2.70 (.66)	2.59 (.59)	2.16 (.52)	.007 A+B, C vs Axis I
Work	2.86 (1.62)	3.01 (1.80)	2.07 (.50)	.94
Social and leisure	3.18 (1.18)	2.96 (1.06)	2.44 (.91)	.06
Extended family	2.29 (.60)	2.00 (.51)	1.95 (.68)	.05
Marital, partnership	2.21 (.56)	2.59 (.78)	2.14 (.63)	.10
Parental	2.00 (.66)	1.91 (.58)	1.52 (.67)	.14
Family unit	2.17 (.96)	2.21 (.95)	2.10 (.81)	.94

As to psychopathology, the Cluster A and B PDs criteria sum scores were significantly higher in the Cluster A+B group compared to the two other groups, and both cluster A+B and cluster C group had significantly higher scores on cluster C criteria sum score than the Axis I group (Table 1). The Cluster A+B group had significantly lower GAF-score and higher PSI score than the two other groups which did not differ from each other. Mental problems had started significantly earlier in the PD groups compared to the Axis I-group. The SAS Overall adjustment was significantly poorer in the cluster A+B and cluster C groups, compared to the Axis I group.

The diagnostic distribution of PDs and Axis I disorders in the three groups at baseline and follow-up are given in Table 2. The mean number of PDs diagnosed in cluster A+B was 2.2, and in cluster C 1.3 per patient, and the most common PDs were avoidant, borderline, and dependent. One PD was observed in 37 patients (51%), two PDs in 20 (28%), and three PDs in 15 patients (21%). Fifty-four (75%) of the PD patient had at least one co-morbid Axis I-disorder.

**Table 2:** Diagnostic distribution of the baseline and follow-up samples.

	**Baseline**			**Follow-up**		
	**Cluster A+B **(n = 39)	**Cluster C **(n = 33)	**Axis I **(n = 19)	**Cluster A+B **(n = 18)	**Cluster C **(n = 21)	**Axis I **(n = 11)

**Personality disorders (DSM-III-R)**	N (%)	N (%)	N (%)	N (%)	N (%)	N (%)
Paranoid	14 (36)	-	-	7 (39)	2 (10)	1 (9)
Schizotyp	6 (15)	-	-	2 (11)	1 (5)	-
Schizoid	4 (10)	-	-	4 (22)	-	-
Antisocial	4 (10)	-	-	1 (6)	-	-
Narcissistic	0 (0)	-	-	0 (0)	-	-
Histrionic	4 (10)	-	-	0 (0)	-	-
Borderline	19 (49)	-	-	2 (11)	-	-
Dependent	5 (13)	7 (21)	-	1 (6)	3 (14)	-
Avoidant	15 (39)	25 (76)	-	11 (61)	15 (71)	3 (27)
Obsessive-compulsive	5 (13)	8 (24)	-	1 (6)	2 (10)	-
Passive-aggressive	8 (21)	2 (6)	-	1 (6)	2 (10)	-
Mean no of PDs	2.2	1.3	-	1.7	1.2	.4

**Axis I disorders (DSM-IV)**						

Major depression	6 (15)	9 (27)	3 (16)	0 (0)	1 (5)	1 (9)
Dysthymia	5 (13)	11 (33)	5 (26)	4 (22)	4 (19)	1 (9)
Panic disorder	7 (18)	7 (21)	2 (11)	0 (0)	2 (10)	1 (9)
Agoraphobia	5 (13)	7 (21)	2 (11)	2 (11)	2 (10)	2 (18)
Social phobia	9 (23)	8 (24)	2 (11)	2 (11)	4 (19)	1 (9)
GAD*	1 (3)	0 (0)	2 (11)	0 (0)	0 (0)	0 (0)
OCD*	4 (10)	2 (6)	2 (11)	0 (0)	0 (0)	1 (9)
Alcohol dependence	10 (26)	2 (6)	3 (16)	1 (6)	0 (0)	2 (18)
Substance dependence	7 (18)	0 (0)	2 (6)	0 (0)	1 (5)	0 (0)
Bulimia nervosa	4 (10)	1 (3)	0 (0)	0 (0)	1 (5)	0 (0)
PTSD*	0 (0)	1 (3)	0 (0)	0 (0)	0 (0)	0 (0)
Mean no of Axis I disorders	1.5	1.5	1.2	.5	0.7	0.8

*GAD: Generalized anxiety disorders, OCD: Obsessive-compulsive disorder, PTSD: Post-traumatic stress disorder

Axis-I disorders were equally common in the two PD groups (mean 1.5 disorder) and with slightly lower mean (1.2) in the Axis I-disorder group. Depressions, anxiety disorders, and alcohol dependence were the most frequent Axis I diagnoses in all groups.

### QoL in PD patients

**Figure 1:**
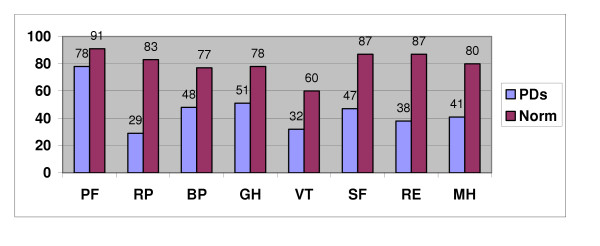
SF-36 mean dimensional scores in personality disorder sample (N = 72) and the age- and gender-adjusted norm sample.

Figure 1. shows that the mean scores on the eight dimensions of the SF-36 of the PD patients at baseline are significantly lower (p < .001 for all) than those of the age- and gender-adjusted norms. The mean difference was least (13 points) for PF, and highest for RF and RE (54 points and 49 points, respectively).

Linear regression analyses showed that control for co-morbid Axis I disorders reduced the PF, GH, VT, SF, and MH scores of the total PD group significantly. Controlling for comorbid cluster C PDs did not influence the SF-36 scores of the cluster A+B PDs to any significant extent, while control for Axis disorders significantly reduced the MH scores in the cluster A+B and cluster C groups.

**Figure 2:**
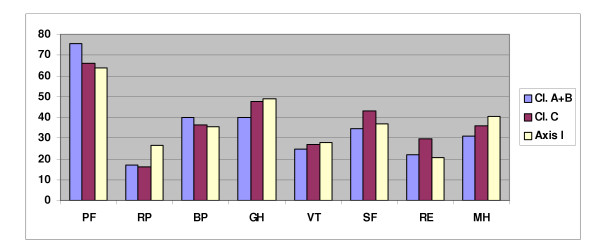
SF-36 mean dimensional scores at baseline for Cluster A+B, Cluster C, and Axis I-disorders groups.

No significant differences were found between genders on any of the SF-36 dimensions among the PD patients (data not shown). Both the cluster A+B, the cluster C, and the Axis I group differed significantly from their norms on all eight SF-36 dimensions (data not shown). No significant differences were observed on the eight SF-36 dimensions between the three diagnostic groups (Figure 2).

When we compared the patients with one (n = 37), two (n = 20), and three or more (n = 15) PDs, we did not observe any significant differences in mean MCS and PCS scores.

### Correlation between SF-36 dimensions and other measures

The eight dimensions of SF-36 are regularly divided into the four physical: PF, RP, BP, and GH, and the four mental ones: VT, SF, RE, and MH. In our PD sample the SF-36 mental dimensions had most significant correlations with the psychiatric measures of the GAF, the SCL-90-R PSI, the sum of positive PDs diagnostic criteria, and the dimensions of the SAS (Table 3). The SF-36 MH had a significant correlation to most of these measures, followed by VT and SF. The physical dimensions of the SF-36 had less frequently a significant correlation to the psychiatric measures.

**Table 3:** Correlation of SF-36 dimensions with Global Assessment of Functioning (GAF), Personality Severity Index (PSI), and dimensions of Social Adjustment Scale (SAS).

	**PF**	**RP**	**BP**	**GH**	**VT**	**SF**	**RE**	**MH**
GAF	.12	.32**	.02	.32**	.26*	.36**	.30**	.40**
Total no of positive PD criteria	.03	-.11	.02	-.27**	-.14	-.18	-.05	-.26**
SCL-90-R PSI	-.22*	-.17	-.12	-.26*	-.25*	-.34**	-.19	-.38**
SAS Overall	-.38**	-.31**	-.23*	-.34**	-.42**	-.45**	-.30**	-.45**
SAS Work	-.17	-.22*	-.10	-.16	-.44**	-.31*	-.26*	-.41**
SAS Social and leisure	-.30**	-.35**	-.24*	-.32**	-.43**	-.39**	-.29**	-.37**
SAS Extended family	-.20	-.18	-.23*	-.21	-.26*	-.32*	-.28*	-.43**
SAS Marital/partner	-.18	.01	-.04	-.18	-.24	-.08	-.05	-.10
SAS Parental	-.04	-.06	.06	.02	-.07	-.09	-.07	-.04
SAS Family unit	-.38**	-.22	-.26*	-.20	-.37**	-.31*	-.27*	-.35**
Sum significant correlation	4	4	4	4	6	7	6	8

* Correlation is significant at the .05 level (two-tailed), ** Correlation is significant at the .01 level (two-tailed)

The SAS overall adjustment and the SAS social and leisure functioning had significant correlations to all the SF-36 dimensions, while the SAS marital/ partnership and the SAS parental functioning had none. The SAS work, extended family, and family unit fell in between.

### What changes in the QoL of PD patients can be observed from baseline to follow-up two years later?

Although quite intensive search for patients was done for the follow-up examination, only 50 patients (53%) of the 91 patients who rated themselves on the SF-36 complied. The distribution of patients were cluster A+B group (n = 18), cluster C (n = 21), and Axis I-disorder group (n = 11). Due to small sample sizes, the Axis I disorder was dropped from further analysis and the two PD groups were pooled as to the study of changes after treatment. The 39 PD patient with SF-36 ratings both at baseline and follow-up were compared to the 33 PD patients only seen at baseline. The diagnoses at follow-up are shown in Table 2, and among the non-compliant patients those with borderline PD, and alcohol and substance dependence were over-represented.

Few significant differences were observed between compliant and non-compliant PD patients at follow-up (Table 4). In particular, no significant differences of the eight SF-36 dimensions were observed between the compliers and non-compliers at baseline. The compliers had significantly more depressive disorders and cluster C PDs at baseline. All of those who terminated treatment without the consent of their therapist (N = 22) were in the non-compliant group. The non-compliant patients also had a significantly longer mean duration of treatment. The mean treatment time for the PD patients attending follow-up was 16.6 months (SD 5.9), median 18 months, and range 4 to 24 months, and the mean follow-up time since treatment termination was 9.8 months (SD 6.4), median 10.4 months, and range 0 to 26 months. The majority of the patients had weekly individual psychotherapy, although a small proportion also had group psychotherapy in addition. Drug treatment was given to 20 patients of the 39 patients, and to 20 of the 33 non-compliers (ns). Among those seen at follow-up, had 15 got antidepressive and 5 antipsychotic medication, in addition to psychotherapy.

**Table 4:** Demographic, psychopathological, and treatment features at baseline for patients with personality disorders with (N = 39) and without (n = 33) follow-up examination.

**Variable**	**Follow-up + **(n = 39)	**Follow-up - **(n = 33)	**p**
Age (mean, SD)	37.9 (11.6)	33.3 (9.9)	.08

Gender (n, %)			.50
Male	17 (44)	17 (52)	
Female	22 (56)	16 (48)	

Relationship (n, %)			.56
Paired	18 (46)	13 (39)	
Non-paired	21 (54)	20 (61)	

Basic education level (n, %)			.64
≤ 9 years	11 (29)	13 (40)	
10 – 12 years	13 (34)	10 (30)	
≥ 13 years	14 (37)	10 (30)	

≥ 1 cluster A PDs	13 (33)	6 (18)	.15

≥ 1 cluster B PDs	10 (26)	15 (45)	.08

≥ 1 cluster C PDs	35 (90)	22 (67)	.02

Mean (SD) of PD cluster criteria			
Total	34.8 (17.9)	34.8 (14.3)	.99
Cluster A	9.5 (8.7)	7.7 (5.1)	.28
Cluster B	7.6 (9.3)	13.1 (10.3)	.02
Cluster C	17.6 (8.9)	14.0 (7.2)	.07

≥ 1 depressive disorder	19 (49)	7 (21)	.02

≥ 1 anxiety disorder	17 (44)	16 (49)	.68

≥ 1 substance use disorder	9 (23)	11 (33)	.33

Comorbid Axis I disorder (n, %)			.79
No	9 (23)	9 (27)	
Yes	30 (77)	24 (33)	

GAF (mean, SD)	46.0 (9.4)	45.7 (9.9)	.91

PSI (mean, SD)	1.5 (.9)	1.6 (.7)	.85

SAS Overall (mean, SD)	2.7 (.6)	2.6 (.6)	.86

SF-36 (mean, SD)*			
Physical Functioning	79.4 (19.1)	76.7 (22.5)	.72
Role Functioning	31.4 (34.3)	25.8 (36.2)	.27
Bodily Pain	47.6 (28.9)	48.1 (26.7)	.71
General Health	51.4 (23.5)	50.5 (20.39	.99
Vitality	35.0 (19.6)	29.1 (18.6)	.16
Social Functioning	45.2 (28.5)	48.9 (21.5)	.60
Role Emotional	42.7 (39.7)	32.3 (31.7)	.35
Mental Health	42.5 (23.2)	38.3 (19.5)	.52

No of sessions (mean, SD)*	16.6 (5.9)	18.8 (26.9)	.01

Termination without consensus (n, %)	0 (0.0)	22 (67)	< . 001

Treated by specialist (n, %)	14 (36)	16 (49)	.28

Additional drug treatment (n, %)	20 (51)	20 (61)	.43

*Mann-Whitney tests

In the 39 PD patients who complied at both baseline and follow-up, significant improvement was seen in the RF and MH dimensions of the SF-36, while considerable, but non-significant changes were observed for BP and SF (Table 5).

**Table 5:** Changes from baseline to follow-up in patients with personality disorders (n = 39).

**Measure**	**Baseline **Mean (SD)	**Follow-up **Mean (SD)	**P**
**SF-36**			
Physical Functioning	79.4 (19.2)	76.8 (24.6)	.95
Role Physical	31.4 (34.3)	51.3 (38.5)	.01
Bodily Pain	47.6 (28.9)	57.5 (25.6)	.06
General Health	51.4 (23.5)	56.0 (26.6)	.22
Vitality	35.0 (19.6)	36.3 (21.4)	.70
Social functioning	45.2 (28.5)	53.5 (28.7)	.09
Role-emotional	42.7 (39.7)	41.9 (38.0)	.89
Mental Health	42.5 (23.2)	50.1 (22.3)	.03

Global Assessment of functioning	46.0 (9.4)	54.6 (9.6)	< .001

Total no of PD criteria	34.8 (17.9)	25.7 (11.5)	< .001

SCL-90-R PSI	1.52 (.86)	1.30 (.80)	.035

**Social Adjustment Scale (SAS)**			
Overall adjustment	2.66 (.63)	2.42 (.62)	.007
Work	2.76 (1.49)	2.36 (1.39)	.20
Social and leisure	3.17 (1.22)	2.87 (1.11)	.045
Extended family	2.05 (.52)	1.97 (.50)	.34

Both professional-rated measures the GAF, and the mean total number of PD criteria, showed significant improvement. Among the patient-rated measures significant better results at follow-up were found for the SCL-90-R PSI, the SAS overall adjustment, and the SAS social and leisure scales.

## Discussion

The main findings of this study of mainly co-morbid PD patients treated at an ordinary POC, was that the QoL on both the physical and mental SF-36 dimensions was significantly lower than that of an age- and gender-adjusted general population sample. According to our knowledge, ours is the first report on QoL-data in such PD patients at a POC. This finding is in accordance with QoL studies of PD patients in the general population [11,13], and correspond to findings of clinical studies of patients with anxiety disorders, depression, schizophrenia, and substance dependence [27-30]. However, the SF-36 dimension mean scores of our PD sample are lower than those reported for these diagnoses, and for co-morbid disorders [31]. In our sample we did not find any significant differences between the SF-36 dimension scores of the cluster A+B, cluster C, or Axis I groups, and all groups had significantly lower scores on all dimensions than their age- and gender-adjusted norm groups.

In contrast to the epidemiological study from Australia [12] we did not find worsening of MCS and PCS with increasing number of PDs present in our sample. This could be due to our small samples, but also due to the fact that our patients with 1 PD had considerably lower QoL than in the Australian survey [MCS: 33.7 (SD 10.6) versus 44.4 (SD 12.0), p < .001, and PCS: 43.8 (SD 8.6) versus 46.9 (SD 11.0), p = .03].

Comorbid Axis I disorders explained a significant part of scores of PF, GH, VT, SF, and MH scores of the total PD group. This is in accordance with the findings of the Australian study [12].

We found that the SF-36 dimensions had variable associations with established psychiatric measures. As expected the SF-36 MH was most strongly associated with the psychiatric measures, but so were also SF and VT. For the SAS we found that overall adjustment and social and leisure activities were significantly correlated to all the SF-36 dimensions. In our PD sample we observed a somewhat different pattern of significant correlations between the GAF and the SF-36 dimensions than reported by Meijer et al. [32] in patients with schizophrenia. Small sample sizes and different diagnostic classes could be the explanation. However, in sum the SF-36 had a considerable association with established psychiatric measures in our PD sample.

For both the patient- and professional-rated psychiatric measures significant changes at follow-up after treatment was observed in the 39 patients who also scored themselves on the SF-36. We cannot say if these changes were related to treatment, and ours is not an outcome study. We wanted to examine if changes in established psychiatric measures were associated with changes in the QoL measured by the SF-36 in the PD patients seen at a POC.

Significant changes at follow-up were found for only two of the SF-36 dimensions, however, one physical (RP) and one mental (MH). While the finding for MH was expected, the change in RP which covered problems with work or other daily activities as a result of physical health was more difficult to explain. The score on that dimension was extraordinarily low at baseline (mean 31.4), and regression towards mean could be a likely explanation. It seemed that only MH of the SF-36 changed in the same way as established psychiatric measures in our study. The SF-36 MH correlated significantly with most of such psychiatric measures, and MH is currently used as a valid measure for mental health in several studies [33].

This result could indicate that the other dimensions of the SF-36 are less valid as measures of changes in mental health of PD patients, or alternatively that most aspects of QoL measured by the SF-36 do not change in PD patients even if established psychiatric measures do.

The main strength of our study was that we were able examine systematically various aspects of the QoL measured by the SF-36 in a clinically relevant sample of PD patients at a POC which is a common setting for such patients in psychiatry.

Our study had a number of weaknesses. The study groups were small with limited statistical power, and there was a considerable risk of type II errors. More significant differences as to the SF-36 dimensions could turn up in larger samples. Although we put considerable efforts into location of patients, we had a lower follow-up rate than we had expected. However, the PD patients who did not show up at follow-up did not differ much from those who did. We cannot, therefore, generalize the discrepancy observed between significant changes in established psychiatric measures and lack of such changes in most of the SF-36 dimensions of PD patients treated at a POC to widely.

The same experienced psychiatrists did all the interviews at baseline and follow-up. Although she was not involved in any treatments, we cannot exclude an expectation bias from her side.

We think that our study has to be considered an exploratory one. Our finding of a generally strongly reduced QoL should be replicated in a PD sample with less comorbid Axis I disorders, although their influence was limited. The same is true for QoL as a valid measure for change in PD patient, since it was not recommended as part of a standard outcome battery and was not used by major treatment studies of PD patients. However, our study confirmed that the SF-36 MH dimension seemed to be a valid psychiatric measure in our PD patient sample.

## Conclusion

In this study of the QoL in PD patients seen at an ordinary POC, we found that the PD patients had significantly lower mean scores on all the SF-36 dimensions compared to age- and gender-adjusted norm data. This is in accordance with the SF-36 measurements of other major diagnostic groups of mental disorders. Although the SF-36 dimensions correlated considerably with established psychiatric measures in our PD patients, they did not show the same significant changes over time as the established measures. The use of QoL measures like the SF-36 as an outcome measure in PD patients is in need of further investigation.

## List of abbreviations

BP: SF-36 Bodily pain

GAF: The Global Assessment of Functioning

GH: SF-36 General health

MH: SF-36 Mental health

PD: Personality disorder

PDs: Personality disorders

PF: SF-36 Physical functioning

POC: Psychiatric outpatient clinic

PSI: Personality severity index of SCL-90-R

QOL: Health-related quality of life

RE: SF-36 Emotional role functioning

RP: SF-36 Physical role

SAS: The Social Adjustment Scale

SCL-90-R: The Symptom Checklist 90-Revised

SF-36: MOS Short Form 36

SF: SF-36 Social functioning

VT: SF-36 Vitality

## Competing interests

The author(s) declare that the have no competing interests.

## Authors' contributions

KN conceived and planned the study, prepared the therapists at Furuset Outpatient Department, did all the psychiatric interviews at baseline and follow-up, and drafted the manuscript. AM helped designing of the study, supervised the statistic calculations, and drafted the manuscript. AAD participated in the design and coordination of the study, and drafted the manuscript. All authors read and approved the final manuscript.

## Pre-publication history

The pre-publication history for this paper can be accessed here:


